# African humid periods triggered the reactivation of a large river system in Western Sahara

**DOI:** 10.1038/ncomms9751

**Published:** 2015-11-10

**Authors:** C. Skonieczny, P. Paillou, A. Bory, G. Bayon, L. Biscara, X. Crosta, F. Eynaud, B. Malaizé, M. Revel, N. Aleman, J. -P. Barusseau, R. Vernet, S. Lopez, F. Grousset

**Affiliations:** 1 IFREMER, Unité de Recherche Géosciences Marines, Z.I. Pointe du diable, BP 70, 29280 Plouzané, France; 2 Université de Lille, CNRS, Université du Littoral Cote d'Opale, UMR8187, LOG, Laboratoire d'Océanologie et de Géosciences, F-59000 Lille, France; 3 LAB, UMR CNRS 5804, Université de Bordeaux, 32271 Floirac, France; 4 Royal Museum for Central Africa, Department of Earth Sciences, 3080 Tervuren, Belgium; 5 SHOM, 29200 Brest, France; 6 EPOC, UMR CNRS 5805, Université Bordeaux, 33615 Talence, France; 7 GEOAZUR, UMR CNRS 7329, Université de Nice-Sofia-Antipolis, 06560 Valbonne, France; 8 CEFREM, Université Via Domitia, 66860 Perpignan, France; 9 IMRS, BP 5055 Nouakchott, Mauritania

## Abstract

The Sahara experienced several humid episodes during the late Quaternary, associated with the development of vast fluvial networks and enhanced freshwater delivery to the surrounding ocean margins. In particular, marine sediment records off Western Sahara indicate deposition of river-borne material at those times, implying sustained fluvial discharges along the West African margin. Today, however, no major river exists in this area; therefore, the origin of these sediments remains unclear. Here, using orbital radar satellite imagery, we present geomorphological data that reveal the existence of a large buried paleodrainage network on the Mauritanian coast. On the basis of evidence from the literature, we propose that reactivation of this major paleoriver during past humid periods contributed to the delivery of sediments to the Tropical Atlantic margin. This finding provides new insights for the interpretation of terrigenous sediment records off Western Africa, with important implications for our understanding of the paleohydrological history of the Sahara.

## Abstract (web summary)

Given the absence of a major river system in the Western Sahara, the source of late Quaternary fluvial sediments along the West African margin remains unclear. Here, the authors present geomorphological data that reveal the existence of a large buried paleodrainage network on the Mauritanian coast.

## Graphical abstract

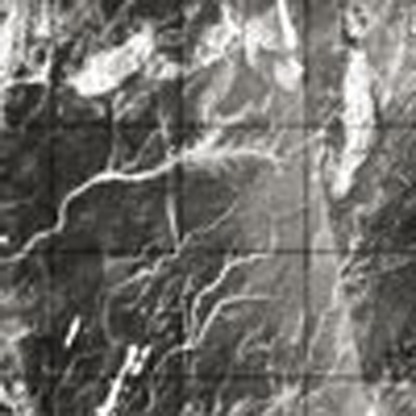

Astronomically forced insolation changes have driven monsoon dynamics and the periodical onset of humid episodes in North Africa over the last few million years, resulting in the ‘greening' of the Sahara and savannah expansion throughout most of the desert at times^1^. These so-called African humid periods (AHPs) were the consequence of a remarkable transformation of the hydrological cycle over North Africa, related to the intensification of the African summer monsoon in response to increased insolation and subsequent northward migration of the Intertropical Convergence Zone (ITCZ). Changes in the position of this rain belt led to the development of important fluvial networks over the Sahara area, which resulted in enhanced freshwater delivery to the surrounding oceans (Fig. 1). Marine sediment records from the Mediterranean and Atlantic margins have provided consistent evidence of monsoon variability in northern Africa since the middle of the Pleistocene^1^. The succession of past AHPs is probably best documented by the deposition of organic-rich sediment layers (sapropels) in the Eastern Mediterranean basin, which has been linked to periods of enhanced discharge from the Nile River back to the Pliocene^2^. The most recent AHP, during the early Holocene, spans from ∼11,700 to 5,000 years BP^3^,^4^,^5^,^6^ and is well recorded in a number of marine sedimentary archives from the Gulf of Guinea to the Northeastern Tropical Atlantic Ocean, the Mediterranean margin and the east of Africa^5^,^6^,^7^,^8^,^9^,^10^.

Off the Western African margin, a region that lies today under the main corridor of Saharan dust plumes, fluvial signals have been identified in deep-sea sediments dated from Marine Isotope stage 5 (ref. 11; that is, ∼120 kyr ago) and the early Holocene^5^,^11^,^12^ (Fig. 2). These sediment layers are characterized by a marked increase in the proportion of fine grain-size mineral particles typical of river-borne material^5^,^11^,^12^, contrasting with the coarser aeolian deposits generally found in this particular region at present^13^, and hence implying enhanced transport of river sediments at these times from the continent to the Mauritanian ocean margin. However, in the present-day climatic context, no permanent fluvial discharge occurs in Western Sahara. In fact, between the Moroccan margin (∼30 °N) and the Senegal River mouth (∼16 °N), only small wadis seasonally reach the Atlantic^14^.

Recently, a spectacular 400-km-long submarine channel system—the Cap Timiris Canyon—has been discovered on the western Sahara margin off Mauritania^15^ (Fig. 1). Large-scale submarine channels generally occur off major river mouths; therefore, the Cap Timiris Canyon was one of the very first channel systems of this kind to be identified offshore a desertic region^15^. These recent findings imply that the Cap Timiris Canyon was connected to a major river system in the past^12^,^15^,^16^,^17^. In fact, a Simulated Topological Network (STN) for potential flow pathways constructed from a digital elevation model also argue for the existence of a large river system in Western Sahara^18^, taking its sources from the Hoggar Highlands and the southern Atlas mountains in Algeria (Fig. 1). This so-called Tamanrasett River valley has been described as a possible vast ancient hydrographic system that would rank twelfth at present among the top 50 largest drainage basins worldwide^18^. Although a putative link between the Tamanrasett paleoriver and the Cap Timiris Canyon has been already suggested previously^12^,^15^,^16^,^17^, direct evidence of any fluvial activity and of a connection to the canyon has never been found on the continent.

In this paper, based on remote-sensing data, we identify the presence of a large paleodrainage network on the arid Mauritanian coast, shallowly buried at present under aeolian sediments. Combined with other geomorpholgical and sedimentary lines of evidence available in the literature, our findings suggest that a major river system was indeed reactivated during some of the humid periods of the last 245 kyrs, thus likely contributing to the delivery of sediments to the Tropical Atlantic margin at those times.

## Results

### PALSAR subsurface geomorphology probing in arid regions

To provide further constraints on the link between past sediment discharges to the Tropical Atlantic margin and fluvial activity in Western Sahara, we have analysed the geomorphology of the Mauritanian coastal area using remote-sensing imagery. We have used the Phased Array type L-band Synthetic Aperture Radar (PALSAR), which is one of the Japanese Advanced Land Observing Satellite remote-sensing instruments. PALSAR is an active microwave sensor using L-band frequency to achieve day-and-night and all-weather land observation. The L-band (1.2 GHz) radar has the capability to penetrate metres of low electrical loss material such as aeolian sand^19^,^20^, and thus to probe the first metres of subsurface geological features in arid areas (see Methods for details). By comparison with the STN^18^, that is, a simulation based on elevation data that provides surface information, the high-performance PALSAR radar has the potential for identifying geomorphological features that may have been buried under shallow aeolian deposits^21^.

### Imaging of the Tamanrasett paleodrainage

Our radar observations of the coastal Mauritania area (Fig. 3a) provide geomorphological evidence for the existence of a paleodrainage system located in the Arguin Bay, between Cap Blanc and Cap Timiris (Figs 3b and 4). This newly identified paleoriver bed is ∼520 km long. Interestingly, it overlaps remarkably well with the coastal section of the course of the Tamanrasett River inferred from the STN (ref. 18; Fig. 4). The reconstruction of the complete paleodrainage was not possible using the PALSAR because of the presence of thick sand dunes, which severely limits the radar detection of underlying sediment structures. However, the branch of the paleodrainage network identified in this study represents a fifth of the total length of the Tamanrasett fossil River highlighted by the STN (ref. 18). Another remarkable feature of our radar data is that the course of the paleoriver is perfectly aligned with paleovalleys identified in the Arguin Basin^22^ (Fig. 3b), as well as with the proximal tributaries of the submarine Cap Timiris Canyon system (Fig. 4). The data presented here hence allow, for the first time, to establish the continuity of this past giant drainage system from the continent (Tamanrasett River) to the shelf (Arguin basin), and then to the bottom ocean (Cap Timiris Canyon).

## Discussion

Previous studies, focused on the morphology and the seismic structures of the Cap Timiris Canyon, have suggested that it may have been active for at least the last 245 kyr (ref. 17) during periods of lower sea-level stand, including during past humid phases of the West African tropical climate^16^. The only well-documented humid phase in the region is the early Holocene AHP, which is characterized by a strong summer insolation (Fig. 2) driving the ITCZ and the associated precipitations to higher latitudes than at the present. At that time, equatorial lakes reached their highest level (Fig. 2) and the present-day Saharan desert was the location of extensive vegetation, animal life and human settlements^3^,^4^,^23^. A recent geochemical study of marine records located off West Africa has estimated a position of the Sahara–Sahel boundary in this area to be ∼21 °N (±3°) during the early Holocene AHP^24^ (Fig. 2). Because the identified branch of the Tamanrasett River lies at 20–23 °N (Fig. 4), enhanced precipitations in the western Sahara region during this AHP could have fed most of the now buried paleodrainage system identified in this study. This hypothesis would be coherent with a recent seismic investigation of the Arguin Basin, which indicates the presence of wadi deltas in the inner part of the basin dated from 8.7 to 6.5 ka (ref. 22), implying significant runoff at that time. In addition, evidence that fluvial sediments were deposited in the Arguin Basin during the mid-Holocene between 11 and 6.5 ka (ref. 22) supports the idea that the Tamanrasett River, at least its coastal part, was active during the last AHP and that it delivered river-borne sediments to the Arguin Basin at that time.

Investigation of sedimentary records from the Eastern Mediterranean Sea off the Nile River has allowed the identification of nine distinct sapropel layers during the last 245 kyr (ref. 1), all occurring during precession-driven summer insolation maxima (Fig. 2). These sapropel deposits and inferred corresponding AHPs are synchronous with the grain-size humidity index maxima recorded on the West African margin for the period for which data are available (the last 120 ka), showing fine fluvial deposits off Mauritania at these times^11^ (Fig. 2). This correlation suggests that Nile floods and Tamanrasett River runoffs were synchronous during the AHPs of the last 120 ka. To date, there is no sedimentological evidence of fluvial deposits recorded off Mauritania for the earlier period. However, assuming that the observed synchronicity between humid phases in Eastern and Western sides of the Sahara held true throughout the 120–245 ka time period of activity of the Cap Timiris canyon, one could infer as many as nine potential periods of runoff for the Tamanrasett River during the last 245 ka. Depending on the extent of the northward migration of the ITCZ and associated rain belt, the span of the reactivated Tamanrasett River section and tributaries has probably varied between the different AHPs. For example, the Saharan vegetation was described as being even more humid during the MIS5e (that is, ∼125 kyr ago), when wooded grassland was predominant throughout the Sahara^1^. This development in the vegetal cover during MIS5e implies that the ITCZ rain belt position reached even higher latitudes than during the Holocene climatic optimum, likely triggering a largest and northernmost reactivation of the Tamanrasett Basin at that time. Additional work would be needed in the future to further constrain the periods of past reactivation of the Tamanrasett River and the variations in the length of its course. However, the lines of evidence reported above suggest that delivery of river-borne material recorded off Mauritania during the early Holocene AHP^5^,^11^,^12^ and previous humid phases (for example, MIS5 (ref. 11)) likely reflects contributions from the Tamanrasett River to the Tropical Atlantic margin at those times.

**Figure 1:**
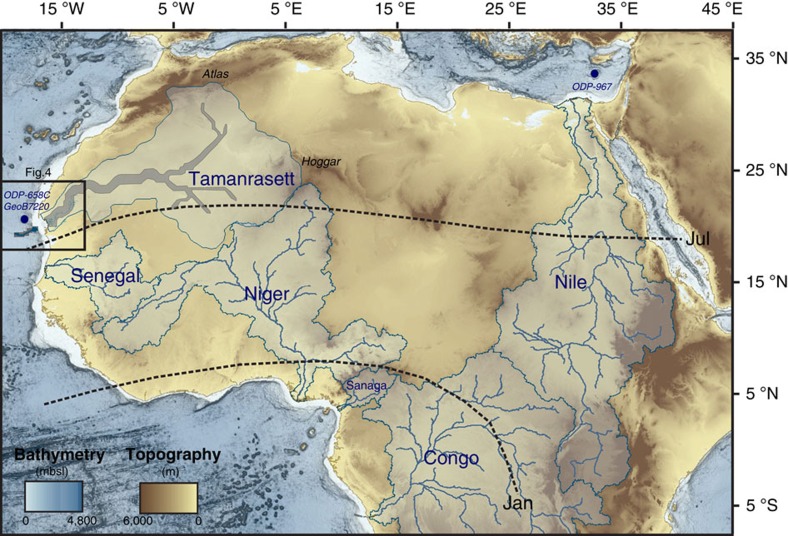
Hydrological context of Africa. Map of the main rivers of the Mediterranean, West African Tropical and Equatorial margins and associated watersheds. The present-day active Nile, Senegal, Niger, Sanaga and Congo rivers watershed are drawn in light blue (adapted from the USGS HydroSHEDS database). The outlines and the main course of the Tamanrasett paleowatershed^18^ are drawn in blue and grey, respectively. The newly identified Tamanrasett paleodrainage (this study) as well as Cap Timiris Canyon^15^ (Fig. 4) are drawn in dark blue. January and July present-day ITCZ positions (dotted lines) as well as GeoB7920 core, ODP658 and ODP967 sites used in Fig. 2 are also plotted.

**Figure 2:**
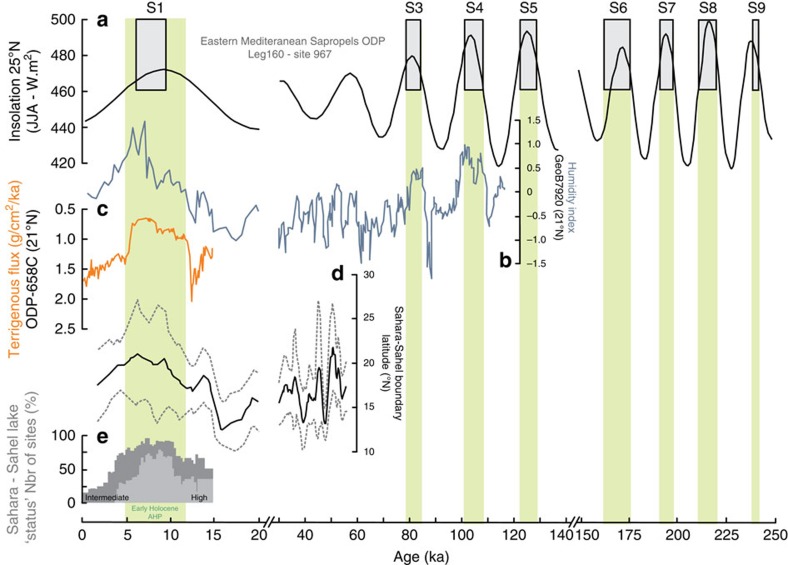
Compilation of North Africa paleoclimate records for the last known period of activity of the Timiris Canyon. (**a**) Sapropels record from core ODP Leg 160 (site 967, Eastern Mediterranean Sea)^38^ together with summer insolation (June, July and August) at 25 °N (ref. 39). The AHPs—identified using the sapropels^1^ (except for the early Holocene AHP)—are highlighted in green. (**b**) Continental humidity index from grain-size measurements of core GeoB7920 (ref. 11; 20.75 °N;18.58 °W), (**c**) Terrigenous Flux^6^ of ODP658 site (20.75 °N; 18.58 °W), (**d**) the estimated latitudinal position of the sedimentary Sahara–Sahel boundary^24^ (black line) with its uncertainty (grey dashed lines). (**e**) Lake level status in East and North African basins^25^.

**Figure 3:**
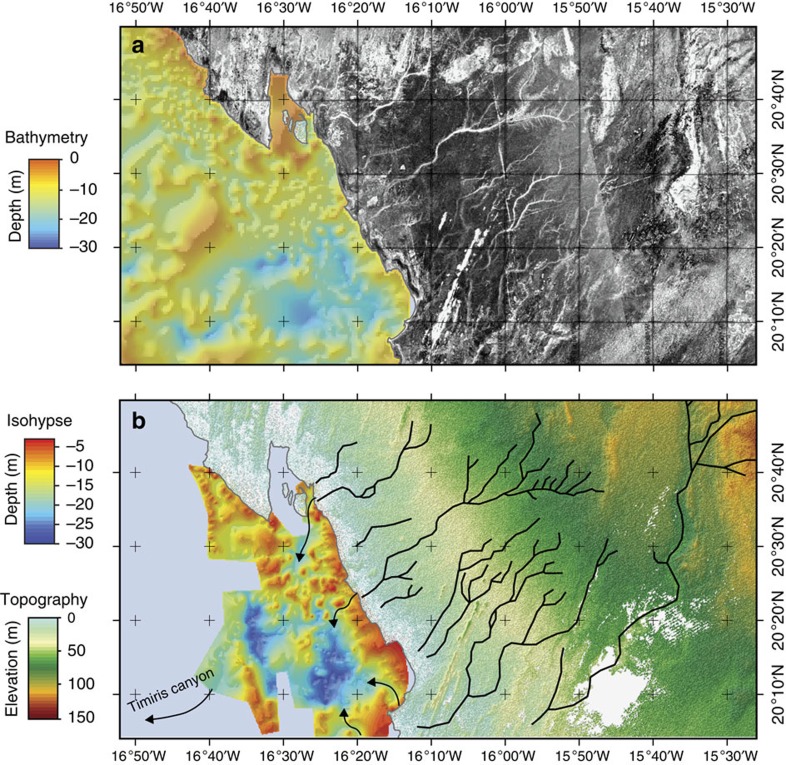
Coastal section of the Tamanrasett paleoriver. (**a**) PALSAR radar observation of the coastal part of the Mauritania. The scale of grey corresponds to the back-scattering power. (**b**) Coastal part of the Tamanrasett paleodrainage (black) identified in the PALSAR image. Isohypse map of the bedrock roof showing the presence of paleovalleys (arrows) of the Arguin Basin^22^.

**Figure 4:**
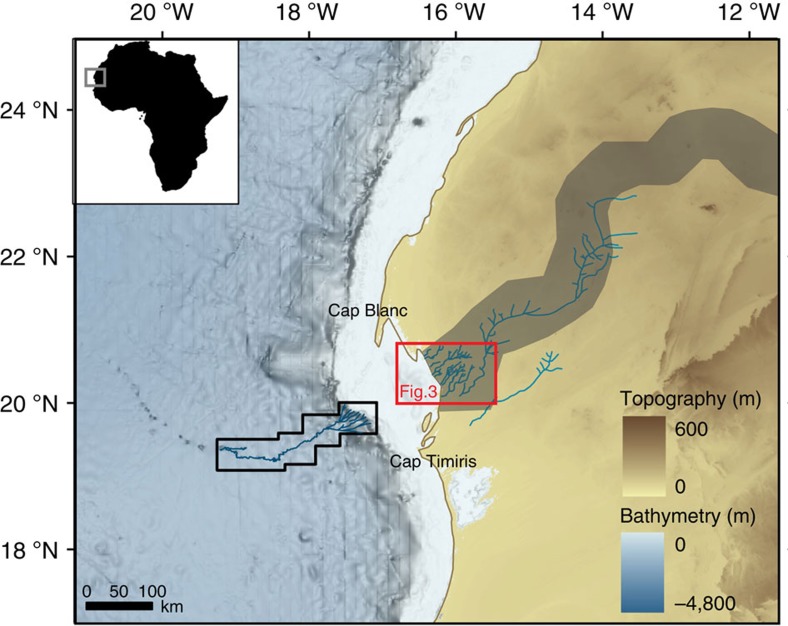
Continuity of the Tamanrasett River-Cap Timiris giant system. Complete identified Tamanrasett paleodrainage (blue), Tamanrasett River water valley as suggested by the Simulated Topological Network^18^ (dark grey band), Cap Timiris Canyon pathway^15^ (dark blue) mapped on the GEBCO bathymetry.

Overall, the identification of the paleodrainage system in the Arguin Bay area is very coherent with the hydrological landscape of Western Sahara, especially during the most recent AHPs. The testimony of a fluvial activity on the Mauritanian coast during recent humid periods provides the missing link between the development of lakes over Algeria and Mauritania^25^, fluvial evidence in Algeria^26^ and riverine signals recorded in the Arguin Basin^22^ and in marine sediments off Mauritania at that times^5^,^11^,^12^. This finding has also major implications for the interpretation of the terrigenous signals recorded during humid periods in marine sediments of the Northeastern Atlantic Tropical Ocean. A marine sediment record often used in the literature as a regional reference for the recent climatic evolution of the West Sahara is the one obtained at the ODP658C site (Fig. 1). This record documents a drastic reduction in terrigenous fluxes—assumed to be entirely of aeolian origin—during the early Holocene AHP^6^ (Fig. 2), and has been used frequently over recent years to illustrate the abruptness of the Saharan environmental response to past insolation changes. The fact that a fraction of the terrigenous material deposited at this site possibly derived from the Tamanrasett River would imply that the dust contribution might have been even lower than inferred during the early Holocene AHP^6^. Our findings therefore support the hypothesis of a major drop in aeolian inputs to the Northeastern Tropical Atlantic at that time, reinforcing the idea that the Saharan environment responds nonlinearly to gradual insolation and hydrological changes^27^. In any case, the likely presence of a fluvial system in the past in the place of present-day major dust sources sheds a new light on the interplay between aeolian and fluvial supplies in the making of the terrigenous signal off West Africa. This finding yet also provides another evidence of the extent of the Saharan wetting during AHPs and should thus provide valuable constraints for numerical simulations of West African climate throughout the late Quaternary.

## Methods

### Synthetic aperture radar subsurface imaging in arid areas

As demonstrated by the Shuttle Imaging Radar (SIR) missions in the early eighties, a radar wave can penetrate dry superficial sediment layers and thus reveal unknown paleohydrological and tectonic structures^28^. L-band (1.2 GHz) radar, in particular, is able to penetrate metres of low electrical loss material such as aeolian sand^19^,^20^. Using the first SIR (SIR-A), the first subsurface imaging results for a site located in the Bir Safsaf region (southern Egypt) was obtained^28^: SIR-A L-band radar revealed buried and previously unknown paleodrainage channels, which were later confirmed during field expeditions^29^,^30^. Subsequently, SIR-C data were used to map subsurface basement structures that control the Nile's course in northeastern Sudan^31^: numerous hidden faults were detected, thus helping to better understand the Cenozoic uplift of the Nubian Swell. Other studies have shown that combining SRTM—Shuttle Radar Topography Mission^32^—topography with SAR images allows to reveal subsurface features under present-day topographic signature. New paleodrainage flow directions have been mapped in the eastern Sahara^33^, allowing a better definition of the drainage lines leading to oases and valleys^33^,^34^. More recently, a major paleodrainage system in eastern Libya, which may have linked the Kufrah Basin to the Mediterranean coast through the Sirt Basin, possibly as far back as the middle Miocene, was mapped^35^,^36^. Synthetic Aperture Radar images from the Japanese Advanced Land Observing Satellite/PALSAR sensor clearly revealed a 900-km-long river system, which starts with three main tributaries in the northeastern Tibesti, northern Uweinat and western Gilf Kebir and ends as a large alluvial fan in the Sarir Dalmah. The sand dunes of the Calanscio Sand Sea prevent deep orbital radar penetration and preclude detailed reconstruction of any possible connection to the Mediterranean Sea, but a 300-km-long link to the Gulf of Sirt through the Wadi Sahabi paleochannel is likely. This link was confirmed by a combined analysis of PALSAR images and SRTM topography^37^: the Kufrah River paleowatershed, at its maximum extent, would have covered more than 400,000 km^2^, representing close to a quarter of the surface area of Libya.

## Additional information

**How to cite this article**: Skonieczny, C. *et al.* African Humid periods triggered the reactivation of a large river system in Western Sahara. *Nat. Commun.* 6:8751 doi: 10.1038/ncomms9751 (2015).

## References

[b1] Larrasona J. C., Roberts A. P. & Rohling E. J. Dynamics of green Sahara periods and their role in hominin evolution. PLoS ONE 8, 10 (2013). 10.1371/journal.pone.0076514 PMC3797788 24146882

[b2] Rohling E. J., Marino G. & Grant K. M. Mediterranean climate and oceanography, and the periodic development of anoxic events (sapropels). Earth Sci. Rev. 143, 62–97 (2015).

[b3] Jolly D. *et al.* Biome reconstruction from pollen and plant macrofossil data for Africa and the Arabian Peninsula at 0 and 6000 years. J. Biogeogr. 25, 1007–1027 (1998).

[b4] Drake N. A., Blench R. M., Armitage S. J., Bristow C. S. & White K. H. Ancient watercourses and biogeography of the Sahara explain the peopling of the desert. Proc. Natl Acad. Sci. USA 108, 458–462 (2011). 21187416 10.1073/pnas.1012231108 PMC3021035

[b5] McGee D., deMenocal P. B., Winckler G., Stuut J. B. W. & Bradtmiller L. I. The magnitude, timing and abruptness of changes in North African dust deposition over the last 20,000yr. Earth Planet. Sci. Lett. 371-372, 163–176 (2013).

[b6] deMenocal P. B. *et al.* Abrupt onset and termination of the African Humid Period: rapid climate responses to gradual insolation forcing. Quat. Sci. Rev. 17, 395–409 (2000).

[b7] Revel M. *et al.* 21,000 years of Ethiopian African monsoon variability recorded in sediments of the western Nile deep sea fan. Reg. Environ. Change 14, 1685–1696 (2014).

[b8] Crosta X., Romero O. E., Ther O. & Schneider R. R. Climatically-controlled silice productivity in the eastern Gulf of Guinea during the last 40,000 yrs. Clim. Past 7, 2445–2476 (2012).

[b9] Weldeab S., Lea D. W., Schneider R. R. & Andersen N. 155,000 years of West African monsoon and ocean thermal evolution. Science 316, 1303–1307 (2007). 17540896 10.1126/science.1140461

[b10] Schefuß E., Schouten S. & Schneider R. R. Climatic controls on central African hydrology during the past 20,000 years. Nature 437, 1003–1006 (2005). 16222296 10.1038/nature03945

[b11] Tjallingii R. *et al.* Coherent high- and low-latitude control of the northwest African hydrological balance. Nat. Geosci. 1, 670–675 (2008).

[b12] Zühlsdorff C., Wien K., Stuut J. B. W. & Henrich R. Late Quaternary sedimentation within a submarine channel-levee system offshore Cap Timiris, Mauritania. Mar. Geol. 240, 217–234 (2007).

[b13] Skonieczny C. *et al.* A three-year time series of mineral dust deposits on the West African margin: sedimentological and geochemical signatures and implications for interpretation of marine paleo-dust records. Earth Planet. Sci. Lett. 364, 145–146 (2013).

[b14] Wynn R. B., Weaver P. P. E., Masson D. G. & Stow D. A. V. Turbidite depositional architecture across three interconnected deep-water basins on the north-west African margin. Sedimentology 49, 669–695 (2002).

[b15] Krastel S. *et al.* Cap Timiris canyon: a newly discovered channel system offshore of Mauritania. EOS 85, 42 (2004).

[b16] Antobreh A. A. & Krastel S. Morphology, seismic characteristics and development of Cap Timiris Canyon, offshore Mauritania: a newly discovered canyon preserved-off a major arid climatic region. Mar. Petrol. Geol. 23, 37–59 (2006).

[b17] Wien K. *et al.* Age models for pelagites and turbidites from the Cap Timiris Canyon off Mauritania Turbidite depositional architecture across three interconnected deep-water basins on the north-west African margin. Sedimentology 49, 669–695 (2006).

[b18] Vörösmarty C. J., Fekete B. M., Meybeck M. & Lammers R. B. Geomorphometric attributes of the global system of rivers at 30-minute spacial resolution. J. Hydrol. 237, 17–39 (2000).

[b19] Elachi C., Roth L. E. & Schaber G. G. Spaceborne radar subsurface imaging in hyperarid regions. IEEE Trans. Geosci. Remote Sensing 22, 383–388 (1984).

[b20] Farr T. G., Elachi C., Hartl P. & Chowdhury K. Microwave penetration and attenuation in desert soil: a field experiment with the Shuttle Imaging Radar. IEEE Trans. Geosci. Remote Sensing 24, 590–594 (1986).

[b21] Paillou P. *et al.* Mapping subsurface geology in Sahara using L-Band SAR: first results from the ALOS/PALSAR imaging radar. IEEE J. Select. Topics Earth Observ. Remote Sensing 3, 632–636 (2010).

[b22] Aleman N., Certain R., Barusseau J. P., Courp T. & Dia. A. Post-glacial filling of a semi-enclosed basin: The Arguin Basin (Mauritania). Mar. Geol. 349, 126–135 (2014).

[b23] Gasse F. Hydrological changes in the African tropics since the Last Glacial Maximum. Quat. Sci. Rev. 19, 189–211 (2000).

[b24] Collins J. A. *et al.* Abrupt shifts of the Sahara-Sahel boundary during Heinrich stadials. Clim. Past 9, 1181–1191 (2013).

[b25] deMenocal P. B. & Tierney J. E. Green Sahara: African Humid Periods paces by Earth's orbital changes. Nat. Educ. Knowledge 3, 7 (2012).

[b26] Chorowicz J. & Fabre J. Organization of drainage networks from space imagery in the Tanezrouft plateau (Western Sahara): implications for recent intracratonic deformations. Geomorphology 21, 139–151 (1997).

[b27] Holmes J. A. How the Sahara became dry. Science 320, 752–753 (2008). 18467577 10.1126/science.1158105

[b28] McCauley J. F. *et al.* Subsurface valleys and geoarchaeology of the eastern Sahara revealed by Shuttle Radar. Science 218, 1004–1020 (1982). 17790589 10.1126/science.218.4576.1004

[b29] Schaber G. G., McCauley J. F., Breed C. S. & Olhoeft. G. R. Shuttle Imaging Radar: physical controls on signal penetration and subsurface scattering in the Eastern Sahara. IEEE Trans. Geosci. Remote Sensing 24, 603–623 (1986).

[b30] Paillou P. *et al.* Sub-surface imaging in central-southern Egypt using low frequency radar: Bir Safsaf revisited. IEEE Trans. Geosci. Remote Sensing 41, 1672–1684 (2003).

[b31] Abdelsalam M. G. & Stern R. J. Mapping precambrian structures in the Sahara Desert with SIR-C/X-SAR radar: the neoproterozoic Keraf suture, NE Sudan. J. Geophys. Res. 101, 23063–23076 (1996).

[b32] Farr T. G. *et al.* The shuttle radar topography mission. Rev. Geophys. 45, RG2004 (2007).

[b33] Drake N. A. *et al.* Palaeohydrology of the Fazzan Basin and surrounding regions: the last 7 million years. Paleo3 263, 131–145 (2008).

[b34] Robinson C. A., El-Baz F., Al-Saud T. S. M. & Jeon S. B. Use of radar data to delineate palaeodrainage leading to the Kufra oasis in the eastern Sahara. J. Afr. Earth Sci. 44, 229–240 (2006).

[b35] Ghoneim E. & El-Baz. F. The application of radar topographic data to mapping of a mega-paleodrainage in the eastern Sahara. J. Arid Environ. 69, 658–675 (2007).

[b36] Paillou P. *et al.* Mapping of a major paleodrainage system in Eastern Libya using orbital imaging Radar: the Kufrah river. Earth Planet. Sci. Lett. 277, 327–333 (2009).

[b37] Paillou P., Tooth S. & Lopez. S. The Kufrah paleodrainage system in Libya: a past connection to the Mediterranean sea? C. R. Geosci. 344, 406–414 (2012).

[b38] Emeis K. C., Sakamoto T., Wehausen R. & Brumsack. H. J. The sapropel record of the eastern Mediterranean Sea—results of Ocean Drilling Program Leg 160. Paleo3 158, 371–395 (2000).

[b39] Laskar, *et al.* A long-term numerical solution for the insolation quantities of the Earth. Astron. Astrophy 428, 261–285 (2004).

